# Chemical Composition and Antioxidant Capacity of the Fruits of European Plum Cultivar “Čačanska Lepotica” Influenced by Different Rootstocks

**DOI:** 10.3390/foods11182844

**Published:** 2022-09-14

**Authors:** Antoaneta Trendafilova, Viktoria Ivanova, Boryana Trusheva, Mariana Kamenova-Nacheva, Sava Tabakov, Svetlana Simova

**Affiliations:** 1 Institute of Organic Chemistry with Centre of Phytochemistry, Bulgarian Academy of Sciences, Acad. G. Bonchev Str., bl. 9, 1113 Sofia, Bulgaria; 2 Research and Development and Innovation Consortium, Sofia Tech Park JSC, 111 Tsarigradsko Shosse Blvd., 1784 Sofia, Bulgaria; 3 Department of Fruit Growing, Agricultural University, Mendeleev Blvd., 12, 4000 Plovdiv, Bulgaria

**Keywords:** *Prunus domestica* L., rootstock, fruit, fruit skin, sugars, organic acids, phenolic compounds, antioxidant activity, ^1^H NMR spectroscopy, LC–DAD–ESIMS, HPLC–DAD

## Abstract

We investigated the influence of different rootstocks on the content of sugars, organic acids, and antioxidant phenolic compounds in the whole fruit and fruit skin of the European plum cultivar “Čačanska Lepotica”. ^1^H NMR of the fruit extracts allowed for the identification of sucrose, α- and β-glucose, sorbitol, fructose, and malic and quinic acids, while LC–DAD–ESIMS showed the presence of neochlorogenic and chlorogenic acids, cyanidin-3-*O*-glucoside, cyanidin-3-*O*-rutinoside, peonidin-3-*O*-glucoside, peonidin-3-*O*-rutinoside, hyperoside, isoquercitrin, rutin, and unidentified quercetin-3-diglycoside. The quantitation of the sugars, malic and quinic acids by ^1^H NMR and phenolic compounds by HPLC–DAD revealed that the rootstock significantly influenced the content of the individual compounds in the fruit skin and fruit. The fruit grafted on “Wavit” rootstock was characterized by significant amounts of neochlorogenic acid, peonidin-3-*O*-rutinoside, cyanidin-3-*O*-rutinoside, and sucrose, while the fruit on “GXN-15” was characterized by high levels of sugars, cyanidin-3-*O*-glucoside, and malic and chlorogenic acids. The fruit skins of plums grafted on “Wavit” were the richest in sugars, organic acids, and phenolic compounds. A good correlation was observed between the content of total phenolics (TPC), flavonoids (TFC), anthocyanins (TAC), and individual phenolic compounds in the extracts of the fruit and the fruit skins and their antioxidant capacity (DPPH, ABTS, and FRAP).

## 1. Introduction

Fruits are valuable sources of nutrients, vitamins, minerals, dietary fibers, nonessential phytochemicals, water, and especially of antioxidant compounds. The European plum (*Prunus domestica*) is cultivated in the temperate zones throughout the world. The fruit of this plant is widely used for nutritional, laxative, and digestive purposes and possesses beneficial effects such as hypotensive, hypoglycemic, and hepatoprotective [1]. Plum fruits are a good source of energy in the form of simple sugars, but they do not mediate a rapid rise in blood sugar concentration, possibly because of their high fiber, fructose, and sorbitol content [2]. Plums also contain large amounts of phenolic compounds, mainly neochlorogenic and chlorogenic acids, which may contribute to the laxative action and delay glucose absorption [2]. Anthocyanins and flavonoids, accumulated predominately in the fruit skin, possess potent antioxidant, anti-inflammatory, antidiabetic, and anticancer activities and, therefore, play an important role in neuroprotective actions and cardiovascular-disease prevention [3]. The taste of a plum depends on the relation of sugars and organic acids, while phenolic compounds can affect the quality of fruit’s sensorial/organoleptic attributes (flavor, aroma, and color), as well as nutritional quality. The phenolic content in the plums varies greatly across cultivar type, growing conditions, geographic location, and environmental factors (water and light availability, soil composition, stresses, etc.) [4,5]. There are also reports about the distribution of different bioactive compounds within the edible part of the fruit (flesh and peel) of *Prunus domestica* [6,7,8]. Rootstocks can affect the vegetative growth and yield, as well as the fruit quality. Although fruit quality is mostly a cultivar-associated trait, it has been reported that the rootstocks have significant effects on the fruit quality (size and color) and fruit nutritional quality, including on the production of biologically active compounds [9,10].

Previous studies of the plum cultivars “Čačanska Lepotica”, “Stanley”, and “Jojo” grown in the experimental nursery of the Agricultural University near Plovdiv (Bulgaria) have shown a significant influence of “Wavit”, “Ishtara”, “GF-677”, and “GXN-15” rootstocks on the growth characteristics of the cultivars [11] and the content of macro- and microelements (K, Ca, Mg, Fe, Zn, Cu, Mn, and B) [12], while the content of sugars, polyphenols, anthocyanins, and organic acids in the fruit flesh was found to be more dependent on the genotype than on the rootstock [13]. The content of the individual phenolic compounds in the plum fruit of these cultivars and their antioxidant properties have not been studied yet.

The present study aimed to characterize the metabolite profile of a methanol extract of the fruit of plum cultivar “Čačanska Lepotica”, using two analytical techniques, nuclear magnetic resonance (NMR) spectroscopy and liquid chromatography–diode-array detection–electrospray mass spectrometry (LC–DAD–ESIMS), to study the influence of different rootstocks (“Wavit”, “Janka”, “Ishtara”, “GF-677”, and “GXN-15”) on the accumulation of some primary metabolites (sugars and organic acids) and phenolic compounds (total phenolics, flavonoids, anthocyanins, and individual compounds) in the fruit and the fruit skin and to determine their antioxidant capacity.

## 2. Materials and Methods

### 2.1. Plum Samples

The fruits of plum cultivar “Čačanska Lepotica” grown on “Wavit”, “Janka”, “Ishtara”, “GF-677”, and “GXN-15” rootstocks were collected from trees cultivated in the experimental nursery of the Agricultural University near Plovdiv, Southern Bulgaria, on 28 July 2021. Trees were formed as free-growing crown, with drip irrigation provided, and a sod/mulch system was applied between the rows. The soil in the rows was maintained with herbicides. Pest and disease control, as well as fertilization, followed the local recommendations for commercial orchards (Supplementary Materials Table S1). Part of the fruits was peeled, and the skins and the fruits of each sample were frozen separately and kept at −20 °C until extraction. The extraction was made separately for the whole fruit and for the skin.

### 2.2. Extraction

Frozen fruits and plum skins were left to defrost at room temperature and homogenized apart in a laboratory blender. Approximately 15 g of the fruit mash or fruit skin was weighted, transferred to an Erlenmeyer flask, and mixed with 20 mL of methanol. The extraction was performed twice for 20 min in an ultrasonic bath, at room temperature. The mixture was filtered, and the filtrates were combined and adjusted to 50 mL with methanol. For NMR analysis, 5 mL of each methanol extract was evaporated to dryness under reduced pressure and protected from light. All extracts and samples were kept in the fridge prior to the analysis.

### 2.3. Qualitative and Quantitative NMR Analysis of Sugars and Organic Acids

The ^1^H NMR spectra were recorded on a Bruker Avance NEO 600 spectrometer (Biospin GmbH, Rheinstetten, Germany) at 298.0 ± 0.1 K. ^1^H spectra in D_2_O (zg30 pulse sequence) were acquired by using 64 scans, 32 K data points, an acquisition time of 5.15 s, and a relaxation delay of 30 s.

For quantitative analysis, the extract (10 mg) was dissolved in 0.5 mL 1 M KH_2_PO_4_ buffer in D_2_O (pH 6.0) containing 0.01% sodium trimethylsilyl propionate (TSP-d_4_) and isonicotinic acid in a concentration of 2 mg/mL as standard. Integration was performed manually. The compound quantities in the studied mixture were based on the integral intensities of respective signals for the individual compounds without overlapping. The two-proton multiplet at δ 8.19 of isonicotinic acid was used as the internal standard. Quantitation was made by using the following general Equation (1):

m_x_ = (m_s_ × N_s_ × I_x_ × M_x_)/(I_s_ × M_s_ × N_x_) (1)

where m_x_ is the mass of the compound to be measured; m_s_ is the weighted mass of the standard; and M_s_ and M_x_, I_s_ and I_x_, and N_s_ and N_x_ are the molar masses (in Da), the integrated signal area, and the number of protons for the respective integrated signal of the standard and the compound, respectively. The content of individual compounds was expressed as g/100 g FW (fresh weight).

### 2.4. Determination of Total Phenolic Content

Total phenolic content (TPC) was determined with Folin-Ciocâlteu reagent [14]. The results were expressed as mg gallic acid equivalents (GAE) per 100 g fresh weight (mg GAE/100 g FW).

### 2.5. Determination of Total Flavonoid Content

Flavonoid content (TFC) was measured by using a colorimetric AlCl_3_ method [14]. The results were expressed as mg quercetin equivalents per 100 g fresh weight (mg QE/100 g FW).

### 2.6. Determination of Total Anthocyanin Content

Total anthocyanin content (TAC) was determined by the pH-differential method [15] and expressed as mg cyanidin-3-*O*-glucoside equivalents per 100 g fresh weight (mg CGE/100 g FW).

### 2.7. LC–DAD–ESIMS Analysis

The LC–DAD–ESIMS analysis was performed on a Shimadzu LC-2040C 3D Nexera-i and Shimadzu LCMS 2020 (single quadrupole). Data acquisition and remote control of the LC–DAD–MS system were performed by LabSolutions (Shimadzu Corporation, Japan) software. The DAD recorded the spectra from 190 to 800 nm, and the wavelengths selected for the qualitative analysis of the compounds were at 320 nm (phenolic acid), 360 nm (flavonoids), and 520 nm (anthocyanins). MS: ESI, negative ion mode; ESI, −3.50 kV; scan range, 100–1000 *m/z*; interface temperature, 350 C; desolvation line, 250 C; heat block, 350 C; nebulizing gas flow, 1.5 L/min; and drying gas flow: 15 L/min. MS: ESI, positive ion mode; ESI, +4.50 kV; scan range, 100–1000 *m/z*; interface temperature, 350 C; desolvation line, 250 C; heat block, 350 C; nebulizing gas flow, 1.5 L/min; and drying gas flow, 15 L/min. The column was a 150 × 2.1 mm id, 2.7 μm bead diameter, Raptor C18 (Restek, Bellefonte, USA, NA), and its temperature was maintained at 40 °C. Gradient elution was carried out with a mixture of two solvents: (A) 0.5% (*v*/*v*) of formic acid in water and acetonitrile (B). The following gradient program was performed at 0 min, 98A:2B; at 15 min, 88A:12B; at 25 min, 75A:25B; at 26 min, 5A:95B; at 31 min, 95A:5B; and at 32 min, 98A:B2 for 5 min. The flow rate was 0.3 mL/min, and the injected volume was equal to 5 μL. The concentration of the samples was around 20 mg/mL in methanol. The extracts and mobile phases were filtered through a 0.22 mm membrane filter and then degassed by ultrasonic bath prior to use.

### 2.8. Quantitative Determination of Individual Compounds by HPLC–DAD Analysis

The HPLC analysis was performed on a Shimadzu Nexera-i LC-2040C 3D Plus liquid chromatograph equipped with a photodiode array detector (Shimadzu, Japan). Column and chromatographic conditions were the same as those used for the LC–DAD–ESIMS analysis. The injection volume of the standards and samples was 2 μL. Before injection, samples were filtered through a 0.22 mm membrane filter. The runs were monitored at the following wavelengths: phenolic acids at 320 nm, flavonoids at 360 nm, and anthocyanins at 520 nm. Retention times (Rt) and UV spectra were compared with those of pure standards. Calibration curves at different concentrations were made from neochlorogenic acid (6.25–100 µg/mL, r^2^-0.9992), rutin (4.15–74.40 µg/mL, r^2^-0.9992), isoquercitrin (1.04–18.65 µg/mL, r^2^-0.9967), cyanidin-3-*O*-glucoside (6.25–100 µg/mL, r^2^-0.9993), and cyanidin-3-*O*-rutinoside (15.50–248 µg/mL, r^2^-0.9990) as standards. The quantities of chlorogenic acid, quercetin diglycoside, hyperoside, peonidin 3-*O*-glucoside, and peonidin 3-*O*-rutinoside were assessed from peak areas and calculated as equivalents of neochlorogenic acid, rutin, cyanidin 3-*O*-glucoside, and cyanidin 3-*O*-rutinoside, respectively. All determinations were performed in triplicate. The concentrations were expressed in mg/100 g FW.

### 2.9. Determination of Antioxidant Capacity

#### 2.9.1. DPPH Radical Scavenging Activity

The 1,1-diphenyl-2-picrylhydrazyl radical (DPPH) scavenging activity assay was performed according to the procedure described by Thaipong et al. [16]. The IC_50_ values were obtained by plotting DPPH scavenging percentage of each sample against the concentration.

#### 2.9.2. ABTS Radical-Ion Scavenging Activity

ABTS (2, 2′-azinobis 3-ethylbenzothiazoline-6-sulfonic acid) radical-ion scavenging activity was performed according the procedure previously described by Thaipong et al. [16]. The results were expressed as Trolox equivalents antioxidant capacity (µM Trolox equivalents per 100 g FW), using a calibration curve of different concentrations of Trolox in methanol (100–500 µM).

#### 2.9.3. FRAP Activity

The assay was performed according to Benzie and Devaki [17], with slight modification. The FRAP reagent was freshly prepared by mixing 10 parts of 0.3 M acetate buffer (pH 3.6), 1 part of 2, 4, 6-tri (2-pyridyl)-1, 3, 5-triazine (TPTZ) in 40 mM HCl, and 1 part of 20 mM FeCl_3_·6H_2_O in distilled H_2_O. The reaction was started by mixing 3 mL FRAP reagent with 100 μL of the investigated sample (diluted with MeOH if necessary). The reaction time was 30 min at room temperature in darkness, and the absorbance was measured at 593 nm against a blank. The FRAP value was calculated from a calibration curve of FeSO_4_·7H_2_O standard solutions and expressed as µM Fe^2+^/100 g FW.

### 2.10. Statistical Analysis

Data from all the measurements that are presented in the tables are the mean of three replicates ± standard deviation. Excel software was used for *t*-test and Pearson correlations. Principal component analysis (PCA) was performed by using SIMCA 17 (Sartorius, Göttingen, Germany, EU).

## 3. Results and Discussion

### 3.1. Qualitative and Quantitative Determination of Carbohydrates and Organic Acids by ^1^H NMR

**Figure 1:**
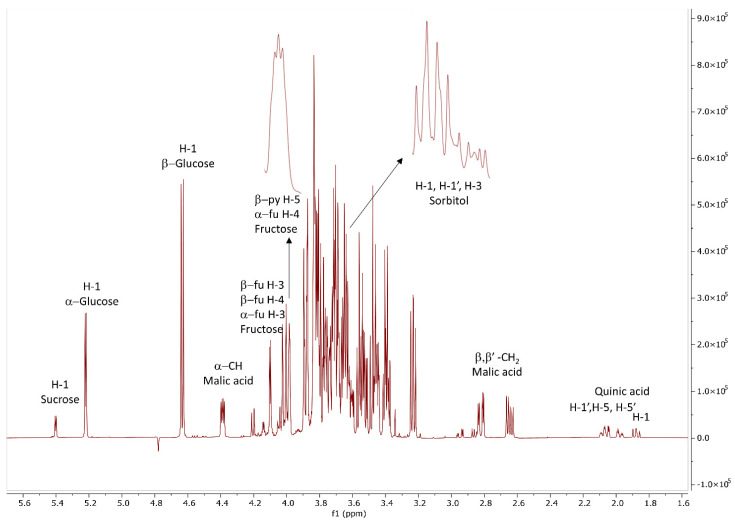
^1^H NMR spectrum of the methanol extract of the fruit skins of the plum cultivar “Čačanska Lepotica”.

^1^H NMR spectroscopy proved to be a valuable tool for identification and quantitation of primary and secondary metabolites of different fruits such as grape, orange, apple juice, cherry, kiwifruits, mango, black raspberry, melon, watermelon, blueberry, plum, and peach [18,19,20,21,22]. The ^1^H NMR spectra of the methanol extracts obtained from the fruit and the fruit skin of plum cultivar “Čačanska Lepotica” grown on “Wavit”, “Janka”, “Ishtara”, “GF-677”, and “GXN-15” showed predominantly the presence of carbohydrates (Figure 1). Two-dimensional NMR experiments and a comparison with the literature data allowed for the unambiguous identification of glucose, sucrose, fructose, and sorbitol [19,20,22,23,24,25,26]. Thus, the resonances of the anomeric protons at 5.40 ppm (J = 3.8 Hz), 5.22 ppm (J = 3.7 Hz), and 4.63 ppm (J = 7.9 Hz) were diagnostic for sucrose, α- and β-glucose, while sorbitol was e recognized by the multiplet at δ 3.64 (3H, H-1, H-1’, and H-3). Fructose was identified from the signals at δ 4.10 (H-3 and H-4, β-furanose; H-3, α-furanose forms) and δ 4.00 (H-5, β-pyranose form; H-4, α-furanose forms) [26]. In addition, the multiplets for malic acid (2.65, 2.82, and 4.39 ppm) and quinic acid (1.88, 1.98, 2.05, and 2.07 ppm) were also identified in the studied extracts [20].

The integral intensities of the selected diagnostic signals (Figure 1) were used for the compound quantitation in the fruit and fruit-skin extracts of the plum cultivar “Čačanska Lepotica” grown on “Wavit”, “Janka”, “Ishtara”, “GF-677”, and “GXN-15” rootstocks by using isonicotinic acid as internal standard (Table 1). The amount of glucose was obtained as the sum of the integration of the α- and the β-anomeric protons. The α-/β-glucose ratio was 1.80 and corresponded to the distribution of both isomers of D-glucose in water at 25 °C. The fructose quantitation was made by using the signals at δ 4.00 and 4.10, taking into account the tautomeric equilibrium at this temperature as 66.85:23.68:6.36:2.53:0.58 in % of the different forms (β-pyranose, β-furanose, α-furanose, α-pyranose, and the keto- forms) [26]. The total sugar content (TSC) was calculated as a sum of the content of sucrose, glucose, fructose, and sorbitol.

The TSC in the whole fruit ranged from 10.12 ± 0.02 to 12.16 ± 0.04 g/100 g FW. The highest TSC was detected in fruit grown on “GXN-15” rootstock. The TSC in the fruit from trees grown on different rootstocks was significantly different (*p* < 0.05), with the exception of those grafted on “Wavit” and “GF-677”. Among individual compounds, glucose was the main component and accounted for 33.6% of TSC on average, followed by sorbitol (30.0% of TSC on average), fructose (23.7% of TSC on average), and sucrose (12.7% of TSC on average). The fruits grown on “Wavit” rootstock were the richest in sucrose (1.60 ± 0.02 g/100 g FW), while those grown on “GXN-15” contained the highest amounts of glucose, fructose, and sorbitol (4.09 ± 0.04, 2.92 ± 0.02, and 3.65 ± 0.07 g/100 g FW, respectively). Fruit grafted on “Wavit”, “Janka”, “GF-677”, and “Ishtara” contained almost equal amounts of glucose (*p* > 0.05). The TSC determined in this study was similar to that found for Čačanska Lepotica” grown in the farms near Čačak (10.18–11.15%) and Belgrade (9.7–14.7%) in Serbia [9,27] and near Ljubljana, Slovenia (9.3–11.1%) [28], but differed from that published recently for the fruits collected from the same place in 2018/2019 [13]. The higher TSC of the fruits collected in 2021 could be explained by the maturity stage and/or annual weather conditions [6,29,30]. Our results showed that monosaccharides are the predominant soluble carbohydrates in fruit, with glucose as the major component, and were in accordance with previous findings for “Čačanska Lepotica” [9,27,28] and other cultivars of *Prunus domestica* [2,6,9,19,27,29]. It is worth mentioning that the contents of sucrose, glucose, fructose, and sorbitol differed from those published recently for the plum cultivar “Čačanska Lepotica” collected from the same place [13]. The authors reported higher amounts of sucrose (1.9–2.3 g/100 g FW) and lower content of sorbitol, glucose, and fructose (2.2–2.7, 1.4–1.7, and 1.0–1.2 g/100 g FW, respectively). The observed differences could be explained by the different maturity stage and/or annual weather conditions [9,29]. Usenik et al. also described that ripening increased the glucose/sucrose and fructose/sucrose ratios due to a lowered concentration of sucrose in fruit [6]. The presence of relatively high content of sorbitol in the studied samples makes these fruits more preferable in special diets as a substitute for glucose and as a remedy for gastrointestinal problems [31].

The TSC in the fruit skin ranged from 8.92 ± 0.02 to 12.45 ± 0.12 g/100 g FW. The highest TSC was detected in the skin of the fruit grafted on “Wavit” rootstock, while the skins from the fruit grown on “Ishtara” and “GXN-15”, and “Janka” and “GF-677” did not differ significantly in their TSC (*p* > 0.05, *t*-test). Among individual compounds, glucose was the main component (38.0% of TSC on average), followed by sorbitol (28.2% of TSC on average), fructose (23.8% of TSC on average), and sucrose (10.0% of TSC on average). The skins from fruit grown on “Wavit” rootstock were the richest in all individual sugars (Table 1). The content of sucrose was almost the same regardless of the rootstock. The skins from fruit grafted on “Ishtara” and “GXN-15” contained almost equal amounts of glucose and sorbitol.

A comparison of the fruit and the skins of the fruit grown on one and the same rootstock showed significant differences (*p* < 0.05, *t*-test) in the TCS and the content of individual sugars, with the exception of the content of glucose in fruit skin and fruit on “Ishtara” rootstock. The TSC was higher in fruit compared to skin, with the exception of the plums grown on “Wavit” and “Janka” rootstocks. The content of sucrose was 1.1–1.5 times higher in the fruit in comparison with fruit skins, regardless of the rootstock. On the contrary, the fruit skins were richer in glucose, with the exception of those grown on “GXN-15”. The observed higher glucose/sucrose and fructose/sucrose ratio in fruit skin than that in the whole fruit is in accordance with a previous study [6].

Two organic acids, malic and quinic acids, were also detected and quantified in the extracts of the fruit and fruit skin of the plum cultivar “Čačanska Lepotica” grown on “Wavit”, “Janka”, “Ishtara”, “GF-677”, and “GXN-15” rootstocks (Table 1). The total organic acid content (TOAC) varied from 0.97 ± 0 to 1.33 ± 0.02 g/100 g FW and did not show any significant difference between the fruit and fruit skin (*p* > 0.05, *t*-test). The fruits grown on “GXN-15” and “Janka” rootstock were the richest in malic and quinic acids, respectively, while the skins from the fruit grafted on “Wavit” rootstock contained the highest amount of both organic acids. The content of malic acid was nearly 2.5 (on average) times higher than that of quinic acid and was in accordance with previous studies of plum cultivars [6,13,19]. The amount of quinic acid was similar to that found for the plum cultivar “Čačanska Lepotica” collected from the same place [13], while that of malic acid was nearly two times higher. This difference could be explained by the different methods used for quantitation (HPLC or NMR) or by the maturity stage [6]. The amount of malic acid in fruit did not differ significantly from that in fruit skin, unlike the content of quinic acid, which was 1.2–1.5 times higher in fruit skin than in the whole fruit. Our comparison of the content of the individual organic acids in the fruit and the skins of the fruit grown on one and the same rootstock showed significant a difference (*p* < 0.05, *t*-test), with exception of those grown on “Janka” (malic and quinic acid) and “GXN-15” (quinic acid).

The TSC/TOAC ratio is a very important indicator for the quality and ripening of the fruit, and according to Forni et al. [31], the TSC/TOAC ratio for the good-quality plums should be higher than 12. The TSC/TAOC ratio in the studied samples varied from 8.5 to 10.8 and was in accordance with previous studies on the “Čačanska Lepotica” cultivar from Serbia [27] and Bulgaria [13] and eight other cultivars of *Prunus domestica* from Bulgaria [32].

### 3.2. Determination of Total Phenolic, Total Flavonoid, and Total Anthocyanin Contents

The total phenolic (TPC), flavonoid (TFC), and anthocyanin (TAC) contents in the methanol extracts of the fruit and the fruit skins of the plum cultivar “Čačanska Lepotica” grown on “Wavit”, “Janka”, “Ishtara”, “GF-677”, and “GXN-15” rootstocks were found to vary in a wide range (Table 2). The fruits contained significant amounts of phenolic compounds with TPC (93.7 ± 1.5–156.1 ± 4.7 mg GAE/100 g FW), while the amounts of flavonoids (11.8 ± 0.2–16.6 ± 0.4 mg QE/100 g FW) and anthocyanins (9.6 ± 0.3–19.2 ± 0.1 mg CGE/100 g FW) were relatively low. As can be expected, anthocyanins and flavonoids were accumulated mainly in the fruit skins, and their amount was 4–6 and 6–12 times higher than that in the whole fruit, respectively. Flavonoids and anthocyanins accounted for 24–30% and 39–48% of the total phenolics in the fruit skins, unlike the whole fruit, where the TFC/TPC and TAC/TPC ratio was less than 13%. In general, the fruit skins were the richest in TPC, TFC, and TAC, and these results are in agreement with previous reports [6,9,33,34,35,36].

It has been found that different rootstocks reflected on the content of phenolic compounds in the fruits from the plum cultivar “Čačanska Lepotica”. Thus, the TPC, TFC, and TAC in the fruits from trees grown on “Wavit” rootstock were 1.7, 1.4, and 2.0 times higher in comparison with fruits grafted on “Ishtara” rootstock. There was no significant difference (*p* > 0.05) in the TPC of the fruits grown on “Janka” and “GF-677” rootstock; in the TFC of the fruits grown on “Janka”, “GXN-15”, and “GF-677” rootstocks; or in the TAC of the fruits grafted on “Janka” and “GXN-15” rootstocks. The obtained results differed from those published recently for the plum cultivar “Čačanska Lepotica” collected from the same place [13]. The authors reported higher values of TPC (205.6–524.3 mg GAE/100 g FW), lower values of TAC (5.1–14.8 mg/100 g FW), and the highest polyphenol content for the fruit flesh from the tree budded on “GF-677” rootstock. The observed differences could be explained by the different annual climatic conditions, the stage of fruit ripening, and the crop-load differences [37].

The fruit skins of the plums grown on “Wavit” rootstock were the richest in TPC (337.3 ± 0.1 mg GAE/100 g FW), TFC (94.4 ± 1.9 mg QE/100 g FW), and TAC (161.9 ± 0.2 mg CGE/100 g FW), followed by the fruit skins of the plums grown on “Ishtara” rootstock with TPC (245.8 ± 4.5 mg GAE/100 g FW), TFC (74.9 ± 3.1 mg QE/100 g FW), and TAC (116.7 ± 1.7 mg CGE/100 g FW). There was no significant difference (*p* > 0.05) in the levels of TPC and TAC of the fruits grown on “GXN-15” and “GF-677” rootstocks and in the levels of TFC of the fruits grown on “Janka”, “GXN-15”, and “GF-677” rootstocks.

### 3.3. Identification of Individual Compounds by LC–DAD–ESIMS

**Figure 2:**
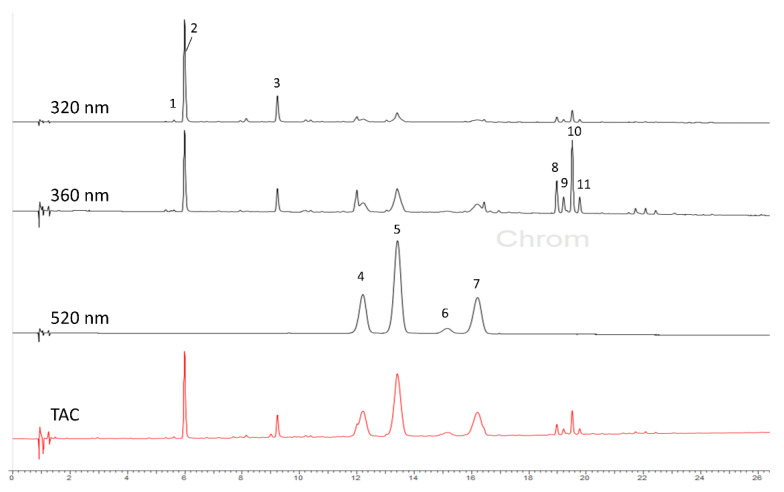
HPLC–DAD chromatogram of the methanol extract of the fruit skins of the plum cultivar “Čačanska Lepotica” grown on “GXN 15” rootstock at 320, 360, and 520 nm. For the peak identification, see Table 3.

The qualitative LC–DAD–ESIMS analysis of the methanol extract of the fruit skin of plums grafted on “GXN-15” rootstock showed the presence of three types of phenolic compounds, namely caffeoylquinic acids, anthocyanins, and flavonol glycosides, which are easily recognized by their characteristic UV absorption maximum at 320, 520, and 360 nm, respectively (Table 3 and Figure 2). The peaks at R_t_ 5.19, 5.82, and 9.05 min (detected at 320 nm) produced [M + H]^+^/[M − H]^−^ at *m*/*z* 355/353 and fragments, characteristic for caffeoylquinic acids [38]. The two most intensive peaks were identified as neochlorogenic acid (3-*O*-caffeoylquinic acid) and chlorogenic acid (5-*O*-caffeoylquinic acid) by comparison with reference standards.

Anthocyanins were observed at 520 nm. Cyanidin-3-*O*-glucoside (R_t_ 12.05 min) and cyanidin-3-*O*-rutinoside (R_t_ 13.25 min) gave [M + H]^+^/[M − H]^−^ at *m*/*z* 449/447 and *m*/*z* 595/593, respectively, and a fragment at *m*/*z* 287 due to the elimination of a sugar moiety. Their presence was confirmed by comparison with reference standards. The peaks at higher retention time (R_t_ 15.00 and 15.94 min) showed [M + H]^+^/[M − H]^−^ at *m*/*z* 463/461 and *m*/*z* 609/607, respectively. The fragment at *m*/*z* 301 in the positive mode, corresponding to a loss of glucoside and rutinoside moiety, revealed the presence of a methoxy group (OCH_3_) in the structure of the aglycone instead of the hydroxyl group in cyanidin structure. Therefore, the two compounds were tentatively identified as peonidin-3-*O*-glucoside and peonidin-3-*O*-rutinoside. To confirm their structures, the methanol extract was separated by solid-phase extraction [39], and the anthocyanin fraction was further analyzed by ^1^H NMR spectroscopy [22,40]. The three-proton singlet at δ 3.99 (OCH_3_), in addition to other characteristic signals for peonidin and sugar moieties, supported the proposed structures (Supplementary Materials Figure S1 and Table S2).

The third group of compounds detected at 360 nm was the group of quercetin glycosides, which were elucidated on the basis of their fragmentation pattern from the aglycone (quercetin, *m*/*z* 303) due to the loss of glycoside moiety. Two peaks at R_t_ 19.03 and 19.62 min displayed [M + H]^+^/[M − H]^−^ at *m*/*z* 465/463, corresponding to quercetin monoglycosides, were assigned as quercetin-3-*O*-galactoside (hyperoside) and quercetin-3-*O*-glucoside (isoquercitrin) by comparison with the authentic standards. The peaks at R_t_ 18.78 and 19.34 min with [M + H]^+^/[M − H]^−^ at *m*/*z* 611/609 revealed quercetin diglycoside isomers. On the basis of their retention time and compared to authentic standard, the second eluting isomer was assigned as rutin. The structure of the compound at R_t_ 18.78 could not be unambiguously identified. All identified compounds, except for quercetin-3-*O*-diglycoside, are described as components of fruit of *Prunus domestica* [1,7,9,41].

### 3.4. Quantitative Determination of Individual Compounds by HPLC–DAD

The content of the individual phenolic compounds (Table 4) in the whole fruit and fruit skins of the plum cultivar “Čačanska Lepotica” grown on “Wavit”, “Janka”, “Ishtara”, “GF-677”, and “GXN-15” rootstocks was determined by HPLC–DAD. The quantification of anthocyanins, caffeoylquinic acids, and flavonol glycosides was performed at 520, 320, and 365 nm, respectively.

Caffeoylquinic acids were the main phenolic components detected in all studied samples. They accounted for 74–82% of the total phenolics. The amount of neochlorogenic acid (3-CQA) was 7–13.8 times higher than that of its isomer chlorogenic acid (5-CQA). The content of 3-CQA varied from 34.08 ± 0.02 to 57.83 ± 0.35 mg/100 g FW, while that of 5-CQA did not exceed 5%. Neochlorogenic acid was the predominating constituent in the flesh of different *Prunus domestica* cultivars, varieties, and hybrids, accompanied by the chlorogenic acid [4,6,7,33,38]. Cinnamic acid was not detected in this study, even though it was described as a dominant component in the flesh of three European plum cultivars (“Čačanska Rana”, “Čačanska Lepotica”, and “Čačanska Najbolja”) grown in the farm near Belgrade [9].

Anthocyanins were the second group in the fruits and accounted 12.5–22.2% of the total phenolics (Table 4). Among them, cyanidin-3-*O*-rutinoside (CRU) was the main component, with a mean value of 8.37 mg/100 g FW. The amount of the other anthocyanins was relatively low, with average values of 1.17, 1.04, and 0.17 mg/100 g FW for cyanidin-3-*O*-glucoside (CGL), peonidin-3-*O*-rutinoside (PERU), and peonidin-3-*O*-glucoside (PEGL), respectively. Cyanidin-3-*O*-rutinoside and cyanidin-3-*O*-glucoside seem to be the characteristic components for the plum fruits, as they are described as the major compounds in many plum cultivars [33,42]. Peonidin-3-*O*-rutinoside and peonidin-3-*O*-glucoside are described as constituents of the fruit of the “Jojo”, “Valor”, “Cacanska rodna”, and “Cacanska najbolja” cultivars [42].

Flavonoids were found to be minor components in the whole fruit (0.67–2.83% of total phenolics). Rutin was the major flavonoid, with average values of 1.98 mg/100 g FW. The second most abundant compound was quercetin diglycoside (mean value of 0.73 mg/100 g FW). The content of hyperoside and isoquercitrin did not exceed 0.1 mg/100 g FW. The obtained results were in agreement with previous reports for different plum cultivars, revealing rutin as the principal flavonol and hyperoside and isoquercitrin as accompanying components [6,7,9,43]. It is worth mentioning that quercetin diglycoside has not been reported as a component of plum fruit so far.

The fruit skin contained 3.3–4.8 times higher amounts of phenolic compounds in comparison with the whole fruit (Table 4). Anthocyanins constituted the main group (44.0–51.1% of total phenolics), followed by caffeoylquinic acids (27.2–34.7%) and flavonol glycosides (15.4–23.2%). Cyanidin-3-*O*-rutinoside (average 62.30 mg/100 g FW), neochlorogenic acid (average 56.29 mg/100 g FW), and rutin (average 25.87 mg/100 g FW) were the major compounds within the three groups of phenolic compounds. Peonidin-3-*O*-rutinoside (25.51 mg/100 g FW), cyanidin-3-*O*-glucoside (18.24 mg/100 g FW), quercetin-diglycoside (17.93 mg/100 g FW), and chlorogenic acid (13.07 mg/100 g FW) were registered in significant amounts, while the amounts of hyperoside, isoquercitrin, and peonidin-3-*O*-glucoside did not exceed 3 mg/100 g FW. Cyanidin-3-*O*-rutinoside has previously been described as the main anthocyanin in the fruit skin of different plum cultivars such as “Jojo”, “Valor”, “Čačanska najbolja”, “Čačanska rodna”, “Haganta”, etc. [6,7,42]. Neochlorogenic acid and rutin were also predominant in the fruit skin of different *Prunus domestica* cultivars and varieties [4,7,33,38].

The results of the *t*-test showed that there were significant differences (*p* < 0.05) in the content of individual phenolics in fruit and skin extracts prepared from the plum cultivar “Čačanska Lepotica” that was grafted from the different rootstocks. Thus, the plums from the trees grafted on “Wavit” rootstock had the highest amounts of neochlorogenic acid, cyanidin-3-rutinoside, peonidin-3-rutinoside, rutin, and quercetin diglycoside in the fruit and skin extracts; and chlorogenic acid, cyanidin-3-*O*-glucoside, peondin-3-*O*-glucoside, and hyperoside in the skin extract. Fruits grown on “GXN-15” rootstock were the richest in chlorogenic acid and cyanidin-3-*O*-glucoside, while those grafted on “CF-677” and “Ishtara” rootstocks were richest in peonidin-3-*O*-glucoside and hyperoside, respectively. The fruit skin obtained from fruits grown on “Wavit” and “Ishtara” rootstocks contained almost equal amounts of isoquercitrin.

### 3.5. Comparison of the Plum Fruit and Fruit Skins Samples

**Figure 3:**
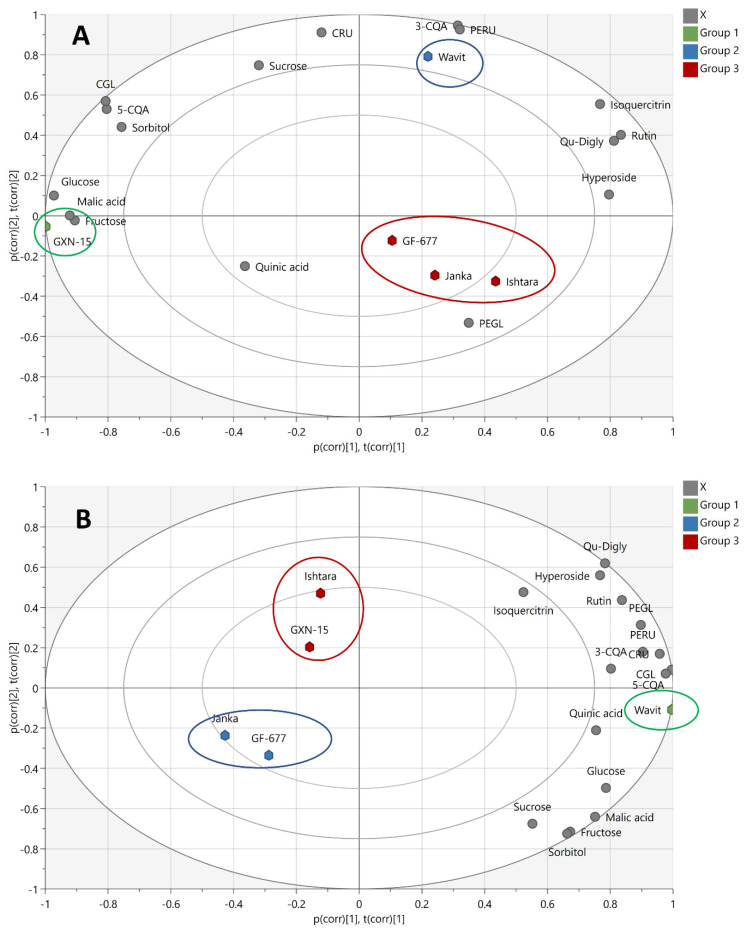
Biplots from PCA of fruit (**A**) and fruit skin (**B**) of the plum cultivar “Čačanska Lepotica” grown on different rootstocks.

PCA was used to establish differences in the chemical compositions of the plum extracts based on the rootstocks. The PCA performed on the content of individual sugars, organic acids, caffeoylquinic acids, anthocyanins, and flavonoids in the plum-fruit extracts showed that the first principal axes accounted for 78.9% of the total variations (Figure 3A).

Two plum-fruit extracts were distinguished from the other plum samples according to different levels of individual compounds. Thus, the sample of fruit grafted on “Wavit” rootstock was separated from the other samples in the positive parts of PC1 and PC2 by its notably higher contents of neochlorogenic acid, peonidin-3-*O*-rutinoside, cyanidin-3-*O*-rutinoside, and sucrose. The high levels of sugars (sorbitol, glucose, and fructose), malic acid, chlorogenic acid, and cyanidin-3-*O*-glucoside separated the sample of fruit grafted on “GXN-15” rootstock from the other samples in the negative sides of PC1 and PC2. The samples of fruit grafted on “Janka”, “Ishtara”, and “GF-677” rootstocks formed the third group, in which the content of peonidin-3-*O*-glucoside was significant.

The PCA performed on the content of individual sugars, organic acids, caffeoylquinic acids, anthocyanins, and flavonoids in the plum-fruit-skin extracts showed that the first principal axes accounted for 85.7% of the total variations (Figure 3B). The sample of fruit skins of plums grafted on “Wavit” rootstock was separated from the other samples by its notably higher content of all individual compounds. The samples of fruit skins of plums grafted on “GF-677” and “Janka” rootstocks formed the second group because of the relatively high content of sugars and organic acids. The third group, consisting of the samples of fruit skins of plums grafted on “GXN-15” and “Ishtara” rootstocks, was characterized by significant amounts of phenolic compounds and the lower content of sugars and organic acids.

### 3.6. Antioxidant Potential

Antioxidant assays (DPPH, ABTS, and FRAP) based on different mechanisms were applied to investigate the antioxidant capacity of the fruit and fruit-skin extracts. The DPPH scavenging assay is widely used for preliminary evaluation of the antioxidant potential of extracts, and it is based on donating electrons from the antioxidants in order to neutralize the DPPH radical. The reaction is accompanied by changing the DPPH color measured at 517 nm, and discoloration acts as an indicator of antioxidant activity [44]. The fruit-skin extracts demonstrated higher DPPH radical scavenging activity (IC_50_ 4.18–5.60 mg/mL) when compared to those obtained from the whole fruit (IC_50_ 6.49–9.40 mg/mL) (Table 5). The extracts obtained from fruits grafted on “Wavit” and “Ishtara” rootstocks were found to be the best DPPH radical scavengers. The fruit and fruit-skin extracts prepared from the fruits of trees grown on “Janka” rootstock showed the highest IC_50_ values (9.40 and 5.60 mg/mL, respectively); therefore, they were the poorest DPPH radical scavengers.

The ABTS assay is another widely used method for the determination of the antiradical scavenging abilities based on the hydrogen-atom-donating tendency of phenolic compounds. The assay measures the capacity of the antioxidants to neutralize the ABTS^●+^, a blue-green chromophore of maximum absorption at 734 nm, whose intensity decreases in the presence of antioxidants [44]. The fruit-skin extracts showed 2–2.5 times higher antioxidant capacity in comparison with the whole-fruit extracts (Table 5). Fruits (whole fruit and skin) grown on “Janka” rootstock were the best ABTS^●+^ scavengers (366.6 and 732.7 µM Trolox/100 g FW, respectively) followed by those grown on “Wavit” rootstock.

The FRAP assay is a typical single-electron-transfer-based method measuring the reduction of the complex of ferric ions (Fe^3+^)-ligand to the intensely blue ferrous complex (Fe^2+^) by means of antioxidants in an acid environment [44]. Antioxidant activity is determined as an increase in absorbance at 593 nm, and the results are expressed as micromolar equivalents of Fe^2+^. The results obtained in the FRAP assay were similar to those by obtained by DPPH and ABTS assays (Table 5). Fruit-skin extracts showed 41.3–74.7% better reducing power than that obtained from the whole fruit. The highest Fe-reducing power was observed for the skins of fruits grown on “Wavit” rootstock, followed by “Ishtara”, “GXN-15”, “GF-677”, and “Janka”. Regarding the whole-fruit extracts, samples from trees grafted on “Wavit”, “GF-677”, and “GXN-15” rootstock showed similar antioxidant capacity, followed by “Janka” and “Ishtara”.

The different antioxidant-activity levels obtained from the DPPH, ABTS, and FRAP assays could be attributed to the difference in the ability of the antioxidant compounds in the extracts to quench ABTS and DPPH free radicals in in vitro systems and to reduce Fe^3+^ or to the applicability of the antioxidant test to hydrophilic and/or lipophilic antioxidants. The antioxidant activities, including the DPPH, ABTS, and FRAP, of different *Prunus domestica* cultivars and varieties have already been reported [4,34,35,36,45,46,47], but it is difficult to compare the results because of differences in the assay procedures or in the solvents used for extraction, etc.

### 3.7. Correlation between Phenolic Compounds and Antioxidant Activity

The correlation between phenolic content (TPC, TFC, TAC, and individual compounds quantified through HPLC–DAD) and antioxidant activities (DPPH, ABTS, and FRAP) was performed with a Pearson’s correlation test (Table 6). The correlation was significant at *p* < 0.001 for all studied variables. A strong correlation was observed between TFC and TAC in the studied extracts and the antioxidant capacity assessed with the DPPH (r = −0.866 and −0.870, respectively) and ABTS (r = 0.950 and 0.932, respectively) tests. Among individual compounds, peonidin-3-*O*-rutinoside (r = −0.891 and 0.956), quercetin diglycoside (r = −0.864 and 0.953), and rutin (r = −0.876 and 0.960) showed the highest correlation with DPPH and ABTS tests, respectively. The antioxidant capacity assessed with the FRAP test showed a significant correlation with TPC (r = 0.968), TAC (r = 0.940), and TFC (r = 0.924), as well as with chlorogenic acid (r = 0.958), cyanidin-3-*O*-glucoside (r = 0.953), and cyanidin-3-*O*-rutinoside (r = −0.934).

## 4. Conclusions

The obtained results revealed differences in the total content of sugars, organic acids, phenolics, flavonoids, anthocyanins, and individual compounds in the fruit skin and the fruit of the European plum cultivar “Čačanska Lepotica” grown on “Wavit”, “Janka”, “Ishtara”, “GF-677”, and “GXN-15” rootstocks. It was found that the fruit skin contained higher amounts of quinic acid, neochlorogenic acid, anthocyanins, and flavonoids and possessed better antioxidant properties measured by DPPH, ABTS, and FRAP assays than the whole plum fruit, while the amounts of sorbitol, fructose, glucose, and malic acid were similar in both fruit and fruit skin. In addition, the rootstocks had a significant influence on the content of nutritive and antioxidant compounds in the plum fruits. Thus, the fruits grown on “Wavit” rootstock produced considerable amounts of neochlorogenic acid, peonidin-3-*O*-rutinoside, cyanidin-3-*O*-rutinoside, and sucrose, while the plum fruit grown on “GXN-15” rootstock contained high levels of sugars (sorbitol, glucose, and fructose), malic acid, chlorogenic acid, and cyanidin-3-*O*-glucoside. Plums grafted on “Wavit” and “GXN-15” rootstocks appeared to have better fruit quality based on their chemical profile (sugars, organic acids, and antioxidants compounds).

## Figures and Tables

**Table 1:** Sugars and organic acids quantified in the fruit and fruit skins of plums growing on different rootstocks by using ^1^H NMR.

Rootstock	Sucrose	Glucose	Fructose	Sorbitol	TSC	Malic Acid	Quinic Acid	TOAC
**Fruit**								
Wavit	1.60 ± 0.02 ^a^	3.62 ± 0.1 ^a–c^	2.48 ± 0.01 ^a^	3.39 ± 0 ^a^	11.09 ± 0.05 ^a^	0.77 ± 0.01 ^a^	0.26 ± 0 ^a–c^	1.02 ± 0 ^a^
Janka	1.35 ± 0.02 ^b^	3.47 ± 0.03 ^a,d^	2.38 ± 0.01 ^b^	2.92 ± 0.01 ^b^	10.12 ± 0.02 ^b^	0.75 ± 0.02 ^a,b^	0.3 ± 0.02 ^a,d–f^	1.05 ± 0.02 ^a,b^
CF-677	1.19 ± 0 ^c^	3.67 ± 0.02 ^b^	2.69 ± 0 ^c^	3.44 ± 0.01 ^c^	11.00 ± 0.01 ^a^	0.83 ± 0 ^c^	0.26 ± 0.01 ^b,d,g,h^	1.09 ± 0.01 ^b^
Ishtara	1.34 ± 0.02 ^b^	3.51 ± 0.02 ^c,d^	2.44 ± 0.01 ^a^	2.98 ± 0 ^d^	10.27 ± 0.01 ^c^	0.73 ± 0.01 ^b^	0.25 ± 0.01 ^c,e^	0.98 ± 0.01 ^c^
GXN-15	1.5 ± 0 ^d^	4.09 ± 0.04 ^e^	2.92 ± 0.02 ^d^	3.65 ± 0.07 ^c^	12.16 ± 0.04 ^d^	0.89 ± 0 ^d^	0.28 ± 0 ^f,h^	1.17 ± 0 ^d^
**Skin**								
Wavit	1.09 ± 0.01 ^a^	4.79 ± 0.21 ^a^	2.98 ± 0.08 ^a^	3.59 ± 0.06 ^a^	12.45 ± 0.12 ^a^	0.94 ± 0.03 ^a^	0.39 ± 0.02 ^a,b^	1.33 ± 0.02 ^a^
Janka	0.99 ± 0.01 ^b,c^	4.06 ± 0.05 ^b^	2.55 ± 0 ^b^	2.8 ± 0.02 ^b^	10.40 ± 0.03 ^b^	0.78 ± 0.01 ^b^	0.35 ± 0.01 ^a,c^	1.13 ± 0.01 ^b,c^
CF-677	1.09 ± 0.01 ^a^	3.79 ± 0 ^c^	2.49 ± 0.02 ^c^	3.17 ± 0 ^c^	10.53 ± 0.01 ^b^	0.78 ± 0.01 ^b,c^	0.32 ± 0 ^d^	1.10 ± 0.01 ^b,d^
Ishtara	0.97 ± 0.01 ^b,d^	3.55 ± 0.04 ^d^	2.01 ± 0.01 ^d^	2.39 ± 0 ^d^	8.92 ± 0.02 ^c^	0.65 ± 0 ^d^	0.32 ± 0 ^e^	0.97 ± 0 ^e^
GXN-15	0.97 ± 0.01 ^c,d^	3.44 ± 0.02 ^d^	2.15 ± 0.02 ^e^	2.54 ± 0.07 ^d^	9.10 ± 0.04 ^c^	0.72 ± 0.05 ^c,d^	0.36 ± 0.04 ^b–e^	1.08 ± 0.04 ^c,d,e^

TSC, total sugar content; TOAC, total organic acid content. All values are expressed as g/100 g FW mean ± standard deviation (*n* = 3). Values with the different letter in the column for fruit and skin are significantly different (*p* < 0.05) (*t*-test).

**Table 2:** Total phenolic (TPC), total flavonoid (TFC), and total anthocyanin (TAC) content in the fruit and fruit skins of plums growing on different rootstocks.

Rootstock	TPC (mg GAE/100 g FW)		TFC (mg QE/100 g FW)		TAC (mg CGE/100 g FW)	
	Fruit	Skin	Fruit	Skin	Fruit	Skin
Wavit	156.1 ± 4.7 ^a^	337.3 ± 0.1 ^a^	16.63 ± 0.4 ^a^	94.4 ± 1.9 ^a^	19.2 ± 0.1 ^a^	161.9 ± 0.2 ^a^
Janka	114.1 ± 4.0 ^b^	203.3 ± 4.4 ^b^	13.3 ± 0.4 ^b,c^	57.9 ± 2.0 ^b^	11.9 ± 0.5 ^b^	79.1 ± 3.0 ^b^
GF-677	120.6 ± 6.3 ^b^	233.5 ± 2.0 ^c^	13.4 ± 0.4 ^b,d^	56.3 ± 2.6 ^b^	13.3 ± 0.4 ^c^	98.1 ± 2.7 ^c^
Ishtara	93.7 ± 1.5 ^c^	245.8 ± 4.5 ^d^	11.8 ± 0.2 ^e^	74.9 ± 3.1 ^c^	9.6 ± 0.3 ^d^	116.7 ± 1.7 ^d^
GXN-15	143.4 ± 1.7 ^d^	229.5 ± 4.6 ^c^	12.9 ± 0.5 ^c,d^	64.5 ± 2.4 ^d^	12.0 ± 0.5 ^b^	101.1 ± 1.9 ^b^

GAE, gallic acid equivalents; QE, quercetin equivalents; CGE, cyanidin-3-*O*-glucoside equivalents. All values are expressed as mg/100 g FW mean ± standard deviation (*n* = 3). Values with the different letters in the column are significantly different (*p* < 0.05) (*t*-test).

**Table 3:** LC–DAD–ESIMS analysis of the methanol extract of the fruit skins of the plum cultivar “Čačanska Lepotica” grown on GXN 15 rootstock.

Peak No.	Compound	Rt (min)	UV (nm)	Molecular Formula	[M + H]^+^/[M − H]^−^ *m*/*z*	Fragmentation Ions *m*/*z*	Method of Identification *
1	Chlorogenic acid–isomer	5.19	325	C_16_H_18_O_9_	355.10/353.05	191 [C_7_H_11_O_6_]^−^	MS
2	Neochlorogenic acid	5.82	325	C_16_H_18_O_9_	355.10/353.05	191 [C_7_H_11_O_6_]^−^	MS, RS
3	Chlorogenic acid	9.05	325	C_16_H_18_O_9_	355.10/353.05	191 [C_7_H_11_O_6_]^−^	MS, RS
4	Cyanidin-3-*O*-glucoside	12.05	519, 280	C_21_H_21_O_11_	449.15/447.05	287 [C_15_H_11_O_6_]^+^	MS, NMR, RS
5	Cyanidin-3-*O*-rutinoside	13.25	519, 280	C_27_H_31_O_15_	595.25/593.15	449 [M-146]^+^, 287 [C_15_H_11_O_6_]^+^	MS, NMR, RS
6	Peonidin-3-*O*-glucoside	15.00	519, 280	C_22_H_23_O_11_	463.15/461.15	301 [C_16_H_13_O_6_]^+^	MS, NMR
7	Peonidin-3-*O*-rutinoside	15.94	519, 280	C_28_H_33_O_15_	609.30/607.15	463 [M-146]^+^, 301 [C_16_H_13_O_6_]^+^	MS, NMR
8	Quercetin-3-*O*-diglycoside	18.78	357, 256	C_27_H_30_O_16_	611.30/609.10	303 [C_15_H_11_O_7_]^+^	MS
9	Hyperoside	19.03	355, 255	C_21_H_20_O_12_	465.13/463.15	303 [C_15_H_11_O_7_]^+^	MS, RS
10	Rutin	19.34	354, 258	C_27_H_30_O_16_	611.25/609.15	465 [M-146]^+^, 303 [C_15_H_11_O_7_]^+^	MS, RS
11	Isoquercitrin	19.62	355, 255	C_21_H_20_O_12_	465.13/463.15	303 [C_15_H_11_O_7_]^+^	MS, RS

* MS, mass spectrometry; NMR, nuclear magnetic resonance; RS, authentic standard.

**Table 4:** Chlorogenic acids, anthocyanins, and flavonoids quantified in fruit and fruit skins of plums growing on different rootstocks by using HPLC–DAD.

Rootstock	3-CQA	5-CQA	CGL	CRU	PEGL	PERU	Rutin	Qu-Digly	Hyperoside	Isoquercitrin
	**Fruit**									
Wavit	57.83 ± 0.35 ^a^	4.17 ± 0.02 ^a^	2.36 ± 0.04 ^a^	11.87 ± 0.02 ^a^	0.02 ± 0 ^a^	3.60 ± 0.07 ^a^	2.62 ± 0 ^a^	1.14 ± 0.02 ^a^	0.05 ± 0 ^a^	0.08 ± 0.01 ^a^
Janka	34.82 ± 0.12 ^b^	3.34 ± 0.09 ^b^	1.07 ± 0.02 ^b^	7.79 ± 0.03 ^b^	0.33 ± 0.01 ^b^	0.82 ± 0.03 ^b^	2.15 ± 0.02 ^b^	0.99 ± 0.01 ^b^	0.03 ± 0 ^b^	0.04 ± 0 ^b^
GF-677	38.83 ± 0.04 ^c^	3.05 ± 0.07 ^c^	1.65 ± 0.03 ^c^	8.60 ± 0.10 ^c^	0.35 ± 0.01 ^c^	0.68 ± 0.07 ^c^	2.38 ± 0.05 ^c^	0.93 ± 0.01 ^c^	0.05 ± 0 ^a^	0.06 ± 0.01 ^c^
Ishtara	36.86 ± 0.10 ^d^	2.56 ± 0.07 ^d^	0.53 ± 0.06 ^d^	5.28 ± 0.01 ^d^	0.10 ± 0.01 ^d^	0.09 ± 0.01 ^d^	1.91 ± 0.01 ^d^	0.60 ± 0.04 ^d^	0.06 ± 0 ^c^	0.06 ± 0.01 ^c^
GXN-15	34.08 ± 0.02 ^e^	4.80 ± 0.08 ^e^	3.00 ± 0.04 ^e^	8.33 ± 0.07 ^e^	0.04 ± 0 ^e^	0.02 ± 0 ^e^	0.92 ± 0.01 ^e^	0.01 ± 0 ^e^	0.02 ± 0 ^c^	0.01 ± 0 ^d^
	**Skin**									
Wavit	69.13 ± 0.12 ^a^	19.95 ± 0.02 ^a^	34.02 ± 0.26 ^a^	87.39 ± 0.75 ^a^	2.21 ± 0.11 ^a^	35.46 ± 0.07 ^a^	34.07 ± 0.04 ^a^	23.10 ± 0.05 ^a^	4.20 ± 0.04 ^a^	1.69 ± 0.03 ^a^
Janka	48.11 ± 0.04 ^b^	9.91 ± 0.03 ^b^	8.74 ± 0.09 ^b^	44.82 ± 0.06 ^b^	0.28 ± 0.03 ^b^	20.42 ± 0.05 ^b^	21.03 ± 0.02 ^b^	13.41 ± 0.06 ^b^	0.66 ± 0.02 ^b^	1.23 ± 0.04 ^b^
GF-677	50.16 ± 0.19 ^c^	10.70 ± 0.02 ^c^	14.01 ± 0.06 ^c^	56.94 ± 0.08 ^c^	0.89 ± 0.06 ^c^	22.57 ± 0.17 ^c^	20.85 ± 0.04 ^c^	13.45 ± 0 ^c^	1.36 ± 0 ^c^	0.30 ± 0.01 ^c^
Ishtara	49.96 ± 0.07 ^c^	12.84 ± 0.01 ^d^	15.77 ± 0.06 ^d^	65.40 ± 0.02 ^d^	1.29 ± 0.05 ^d^	28.61 ± 0.05 ^d^	30.63 ± 0.04 ^d^	21.13 ± 0.01 ^d^	3.11 ± 0.03 ^d^	1.73 ± 0.01 ^a^
GXN-15	64.10 ± 0.16 ^d^	12.01 ± 0 ^e^	18.67 ± 0.21 ^e^	56.96 ± 0.13 ^c^	1.39 ± 0.12 ^d^	20.50 ± 0.19 ^b^	22.77 ± 0.02 ^e^	18.56 ± 0.01 ^e^	3.43 ± 0.01 ^e^	0.72 ± 0.02 ^d^

All values are expressed as mg/100 g FW mean ± standard deviation (*n* = 3). Values with the different letters in the column for fruit and skin are significantly different (*p* < 0.05) (*t*-test).

**Table 5:** Antioxidant potential (DPPH, ABTS, and FRAP) of fruit and fruit-skin extracts obtained from plums growing on different rootstocks.

Rootstock	DPPH IC_50_ (mg/mL)		ABTS (µM Trolox/100 g FW)		FRAP (µM Fe^2+^/100 g FW)	
	Fruit	Skin	Fruit	Skin	Fruit	Skin
Wavit	6.49 ± 0.18 ^a^	4.18 ± 0.02 ^a^	350.65 ± 0.51 ^a^	728.69 ± 4.04 ^a^	2.89 ± 0.10 ^a,b^	6.16 ± 0.63 ^a^
Janka	9.40 ± 0.15 ^b^	5.60 ± 0.04 ^b^	366.56 ± 2.84 ^b^	732.69 ± 2.27 ^a^	2.26 ± 0.05 ^c^	3.49 ± 0.11 ^b^
GF-677	8.86 ± 0.15 ^c^	4.42 ± 0.01 ^c^	354.41 ± 1.17 ^c^	689.94 ± 0.53 ^b^	2.92 ± 0.12 ^a,d^	3.91 ± 0.24 ^b,c^
Ishtara	6.73 ± 0.06 ^a^	4.21 ± 0.02 ^a^	345.94 ± 0.11 ^d^	717.68 ± 1.95 ^c^	1.89 ± 0.03 ^e^	4.58 ± 0.12 ^d^
GXN-15	8.66 ± 0.32 ^d^	5.39 ± 0.01 ^d^	345.97 ± 4.23 ^a,d^	676.98 ± 2.51 ^d^	2.76 ± 0.1 ^b,d^	4.02 ± 0.24 ^c^

All values are expressed as mean ± standard deviation (*n* = 3). Values with the different letters in the column are significantly different (*p* < 0.05) (*t*-test).

**Table 6:** Pearson’s correlation coefficients (r) between phenolic content (TPC, TFC, TAC, and individual compounds) and antioxidant activities (DPPH, ABTS, and FRAP).

	TPC	TFC	TAC	3-CQA	5-CQA	CGL	CRU	PEGL	PERU	Qu-Digly	Hyperoside	Rutin	Isoquercetin	DPPH	ABTS	FRAP
TPC	--															
TFC	0.962	--														
TAC	0.975	0.994	--													
3-CQA	0.826	0.771	0.785	--												
5-CQA	0.987	0.979	0.986	0.794	--											
CGL	0.966	0.938	0.959	0.820	0.982	--										
CRU	0.974	0.993	0.999	0.780	0.982	0.952	--									
PEGL	0.897	0.894	0.918	0.754	0.924	0.961	0.910	--								
PERU	0.962	0.993	0.992	0.757	0.969	0.921	0.993	0.871	--							
Qu-Digly	0.931	0.990	0.981	0.757	0.954	0.912	0.983	0.891	0.981	--						
Hyperoside	0.898	0.919	0.928	0.796	0.928	0.945	0.922	0.966	0.890	0.936	--					
Rutin	0.941	0.994	0.987	0.732	0.959	0.909	0.988	0.876	0.994	0.993	0.907	--				
Isoquercitrin	0.831	0.907	0.872	0.609	0.868	0.793	0.865	0.756	0.897	0.898	0.809	0.917	--			
DPPH	−0.828	−0.866	−0.870	−0.756	−0.817	−0.768	−0.873	−0.692	−0.891	−0.864	−0.759	−0.876	−0.750	--		
ABTS	0.872	0.950	0.932	0.661	0.887	0.810	0.940	0.748	0.956	0.953	0.793	0.960	0.845	−0.856	--	
FRAP	0.968	0.924	0.940	0.789	0.958	0.953	0.934	0.918	0.918	0.892	0.893	0.902	0.829	−0.750	0.798	--

Significant correlation with *p* < 0.001, two-tailed, *n* = 30.

## Data Availability

The dataset of the current study is available from the corresponding authors on reasonable request.

## Supplementary Materials

The following supporting information can be downloaded at https://www.mdpi.com/article/10.3390/foods11182844/s1, Table S1: Fertilize regime and plant protection of the plum trees. Figure S1: 1H NMR spectrum of the anthocyanin enriched fraction obtained from plum fruit skins from plums grafted on “GXN-15” rootstock in CD_3_OD. Table S2: ^1^H NMR spectral data of the anthocyanins in CD_3_OD (600 MHz).
