# Supplementary material for: Chemical Composition and Antioxidant Capacity of the Fruits of European Plum Cultivar “Čačanska Lepotica” Influenced by Different Rootstocks
Source: Foods. 2022 Sep 14;11(18):2844. doi: 10.3390/foods11182844 (PMC9498388; doi:10.3390/foods11182844)
Supplement: Supplementary file 1 [file foods-11-02844-s001.zip › foods-1892388-supplementary.pdf]

SUPPLEMENTARY MATERIAL

Chemical composition and antioxidant capacity of the fruits of European plum cultivar "Čačanska Lepotica" influenced by different rootstocks

Antoaneta Trendafilova[1,*], Viktoria Ivanova[1], Boryana Trusheva[1], Mariana Kamenova-Nacheva[1,2], Sava Tabakov[3], and Svetlana Simova[1]

[1] Institute of Organic Chemistry with Centre of Phytochemistry, Bulgarian Academy of Sciences, Acad. G. Bonchev Str., bl. 9, 1113 Sofia, Bulgaria
[2] Research and Development and Innovation Consortium, Sofia Tech Park JSC, 111 Tsarigradsko Shosse Blvd., Sofia 1784, Bulgaria
[3] Agricultural University, Department of Fruit Growing, Mendeleev Bld., 12, 4000 Plovdiv, Bulgaria
* Correspondence: Antoaneta.Trendafilova@orgchm.bas.bg

Table S1. Fertilization regime and plant protection of the plum trees.

**Fertilize regime**		**Plant protection**	
**Autumn fertilization November**	Nitrogen/NH4NO3/ active substance 12 kg/ha	**Autumn**	Copper hydroxide /Cu(OH)2/ active substance 4 kg/ha
	Potassium/K2SO4/ active substance 30 kg/ha	**Spring**	Copper hydroxide /Cu(OH)2/ active substance 4 kg/ha
	Phosphorus/P2O5/ active substance 50 kg/ha	**Vegetation**	*Bacillus subtilis* QST 713 active substance 10 L/ha
**Spring fertilization late February**	Nitrogen/NH4NO3/ active substance 45 kg/ha		Flonicamid, g/kg 500 active substance 140 g/ha
	Potassium/K2SO4/ active substance 50 kg/ha		Spinetoram, g/kg 250 active substance 500 g/ha
	Phosphorus/P2O5/ active substance 20kg/ha		Chlorantraniliprole 200 g/L active substance 600 mL/ha
			Abamectin g/L 18 active substance 1 L/ha

Isolation, purification, and characterization of anthocyanins in the methanol extract of plum fruit skin by ^{1}H NMR

The isolation and purification of anthocyanins in the methanol extract of plum-fruit skinby ^{1}H NMR was performed by using the procedure described by Rodriguez-Saona and Wrolstad [1]. Briefly, the Discovery®DCS-18 cartridge (500 mg, 3 mL, Supelco, USA) was conditioned by the passing of two column volumes of MeOH and three volumes of acidified deionized H_2O (0.01 % HCl, *v*/*v*). Then a solution of dry methanol extract (50 mg) of plum-fruit skins in deionized H_2O (1 mL) was applied on the cartridge and washed with two column volumes of acidified deionized H_2O to remove compounds not absorbed (e.g., acids and sugars). The cartridge was further washed with two volumes of ethyl acetate to remove phenolic acids and flavonoids. The anthocyanins were eluted with acidified MeOH (0.01 % HCl, *v*/*v*). The methanol was removedin a rotary evaporator under vacuum and protected from light. The

resulting anthocyanin-enriched fraction was further dissolved in CD_3OD. The ^1H NMR, COSY, HSQC, and HMBC spectra were recorded on a Bruker Avance NEO 600 spectrometer (Biospin GmbH, Rheinstetten, Germany). The individual compounds were identified through the comparison of the ^1H NMR data (Figure S1) with those published in the literature [2,3]. The chemical shifts, multiplicity, and the coupling constants of the aglycone and anomeric protons are given in Table S2.

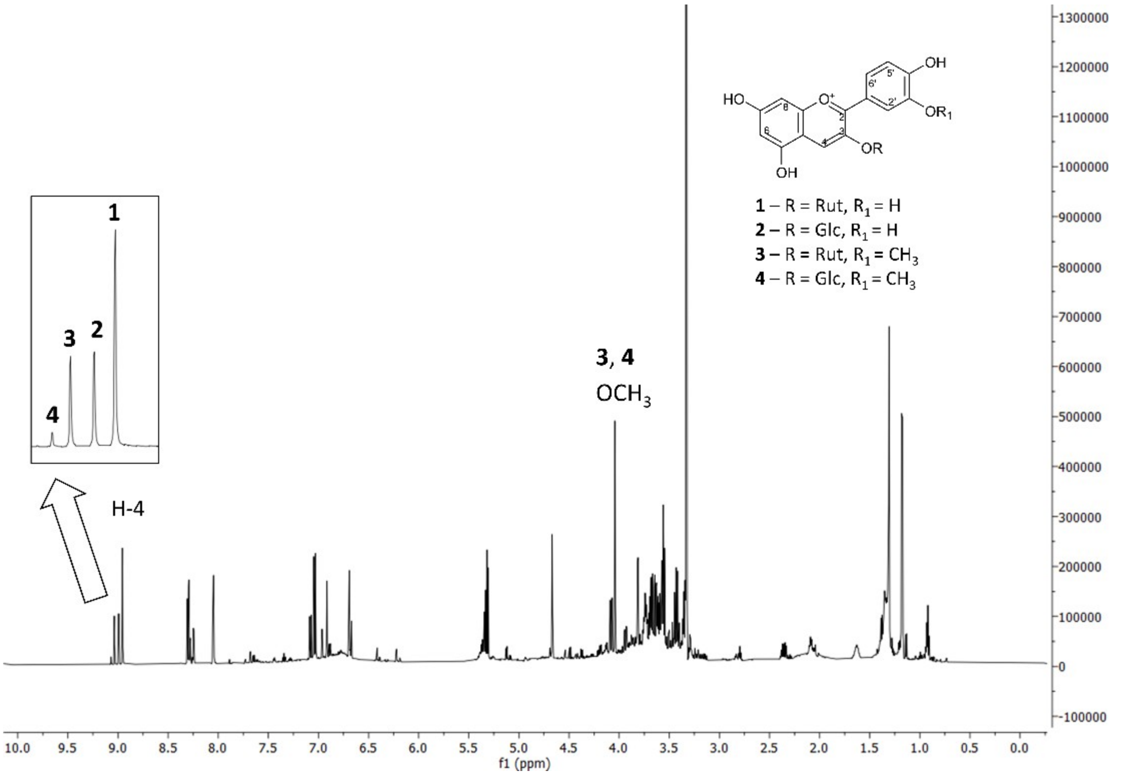

Figure S1. ^1H NMR spectrum of the anthocyanin-enriched fraction obtained from plum-fruit skins from plums grafted on "GXN-15" rootstock in CD_3OD.

Table S2. ^1H NMR spectral data of the anthocyanins in CD_3OD (600 MHz).

H	1	2	3	4
4	8.94 s	8.97 s	9.02 s	9.05 s
6	6.69 d (2.0)	6.67 d (2.0)	6.70 d (2.0)	6.70 d (2.0)
8	6.92 d (2.0)	6.92 d (2.0)	6.96 d (2.0)	6.96 d (2.0)
2′	8.03 d (2.2)	8.04 d (2.2)	8.22 d (2.2)	8.23 d (2.2)
3′	7.02 d (8.8)	7.02 d (8.8)	7.06 d (8.8)	7.06 d (8.8)
6′	8.30 dd (2.2, 8.8)	8.30 dd (2.2, 8.8)	8.28 dd (2.2, 8.8)	8.28 dd (2.2, 8.8)
OCH3	-	-	3.99 s	3.98 s
H-1″ (Glc)	5.30 d (7.6)	5.30 d (7.6)	5.32 d (8.0)	5.32 d (8.0)
H-1‴ (Rha)	4.65 d (1.5)	-	4.65 d (1.5)	-
H-6‴ (Rha)	1.16 d (6.5)	-	1.16 d (6.5)	-

References:

1. Rodriguez-Saona, L.E.; Wrolstad, R.E. Extraction, Isolation, and Purification of Anthocyanins. *Curr. Protoc. Food Anal. Chem.* **2001**, *1*, F1.1.1-F1.1.11, doi:10.1002/0471142913.faf0101s00.

2. Andersen, Ø.M.; Fossen, T. Characterization of Anthocyanins by NMR. *Curr. Protoc. Food Anal. Chem.* **2003**, *9*, F1.4.1-F1.4.23, doi:10.1002/0471142913.faf0104s09.

3. Goulas, V.; Minas, I.S.; Kourdoulas, P.M.; Lazaridou, A.; Molassiotis, A.N.; Gerothanassis, I.P.; Manganaris, G.A. 1H NMR Metabolic Fingerprinting to Probe Temporal Postharvest Changes on Qualitative Attributes and Phytochemical Profile of Sweet Cherry Fruit. *Front. Plant Sci.* **2015**, *6*, 959, doi:10.3389/fpls.2015.00959.
